# Vaccine Hesitancy and Political Populism. An Invariant Cross-European Perspective

**DOI:** 10.3390/ijerph182412953

**Published:** 2021-12-08

**Authors:** Almudena Recio-Román, Manuel Recio-Menéndez, María Victoría Román-González

**Affiliations:** Department of Economics and Business, University of Almería, Carretera de Sacramento s/n, 04120 Almería, Spain; mvroman@ual.es

**Keywords:** vaccine hesitancy, populism, alignment, invariance, social marketing

## Abstract

Vaccine-hesitancy and political populism are positively associated across Europe: those countries in which their citizens present higher populist attitudes are those that also have higher vaccine-hesitancy rates. The same key driver fuels them: distrust in institutions, elites, and experts. The reluctance of citizens to be vaccinated fits perfectly in populist political agendas because is a source of instability that has a distinctive characteristic known as the “small pockets” issue. It means that the level at which immunization coverage needs to be maintained to be effective is so high that a small number of vaccine-hesitants have enormous adverse effects on herd immunity and epidemic spread. In pandemic and post-pandemic scenarios, vaccine-hesitancy could be used by populists as one of the most effective tools for generating distrust. This research presents an invariant measurement model applied to 27 EU + UK countries (27,524 participants) that segments the different behaviours found, and gives social-marketing recommendations for coping with the vaccine-hesitancy problem when used for generating distrust.

## 1. Introduction

Vaccine hesitancy and political populism are propelled by similar motivations: a profound distrust in elites and experts. Political influences are one of the contextual determinants of vaccine hesitancy behaviour [1]. Kennedy [2] found that, in 14 European countries, there was a significant association between the percentages of people in a country who voted for populist parties and of those who believed that vaccines were not important or effective. In the same way, Peretti-Watel et al. [3] found that, in France, those who had voted for a far left or far right candidate, as well as those who abstained from voting, were much more likely to state that they would refuse vaccines. To the best of our knowledge, so far no one has studied systematically the relationship between citizen’s populist attitudes and vaccine hesitancy at an individual level in a large sample of countries. Hence, we aim to expand previous works by studying this underlying link with individual data that come from a large-scale survey that includes all the countries that belong to EU-27 + U.K. In that vein, Europe is the region with the highest level of vaccine hesitancy [4] and populism has recently been on an upward trend [5]. 

Vaccine hesitancy—the reluctance or refusal to vaccinate despite the availability of vaccines—is a global issue that has become more pronounced in recent years in almost every country [6]. One distinctive characteristic of vaccines, when compared with other medicines, is that it works both at individual and community level. Even in countries with high national vaccine uptake rates, if a population subgroup delays in acceptance or refusal of vaccines, the overall immunization strategy (at regional, national or global level) could be in danger [1].

Achieving a quantitative knowledge of the factors associated with anti-vaccination attitudes is challenging due to the “small pockets” problem: the number of people with strong anti-vaccination attitudes represents a small minority of the population. Furthermore, any large-scale survey must be context-specific and valid. The team managing the survey, and the ulterior statistical analysis performed with the collected data, need to prove that scales are suitable in all countries and that their scores and relationships among variables could be compared. Statistically speaking, this issue is called measurement invariance.

Measurement invariance is becoming an increasingly important topic, but not yet in the vaccine hesitancy ground. In July 2021, the terms related to measurement invariance returned about 6337 hits in a Web of Science search (see Appendix A, Table A1 search #10). Vaccine hesitancy also attracted the interest of researchers, yielding about 5225 hits (see Appendix A, Table A1, search #3). For answering the question of to what extent the research about vaccine hesitancy did measurement invariance analysis, we crossed the previous queries and obtained only 2 hits (see Appendix A, Table A1, search #11). Main scales used for measuring vaccine hesitancy, based on scores of latent factors from multiple observed responses, are valid to the extent that the estimated parameters hold in each group under study (v.gr. country, region, etc.). Therefore, there is a gap in the scientific literature about vaccine-hesitancy. 

Social marketing has been long employed in designing, implementing, and evaluating public health programs in the fight against several forms of communicable diseases [7,8,9]. From the marketing perspective, audience targeting and segmentation strategy are the keys to success [10]. To maximize vaccine uptake, better targeting is reached when based on people’s attitudes, values, and observed behaviours [11]. For this purpose, we used invariant measures of two of the main determinants of vaccine hesitancy—distrust and usefulness of vaccines [12]—and political populism ideas in the European citizens for determining how these variables relate to each other in EU27 + UK. Considering the average score that the interviewees in each country had in these three variables, we clustered them to obtain the different segments of European countries attending to the relationship between vaccine hesitancy and political populism. Governments and public health services (either international, national, or regional) could use these results when designing social marketing programs for overcoming hesitancy barriers associated with political populism beliefs, attitudes, and behaviours.

## 2. Populism and Vaccines

Political ideology influences how policymakers address and give solutions to healthcare issues [13]. Recently, many established democracies have experienced a flourishing of populist political movements [14]. Most of them belong to the right-wing political spectrum, but not necessarily. Hence, they do not need to share the same interests but only to fight against the same enemy (“the elite” or “the establishment”) that frustrates and endangers them all (“the people”) [15]. The elite normally refers to mainstream political parties, the media, the upper classes, intellectuals, and, in the territorial scope of this work, the European Union [16]. When applied to healthcare matters, medical populism [17] is based on a distrust of evidence-based policy interventions and the condemnation of technocratic knowledge [18,19,20]. 

Vaccine resistance fits perfectly in populist agendas. High levels of distrust and polarization are fruitful grounds for amplifying dissatisfaction with the elite [21]. Citizen’s feelings about vaccines depend on their perceptions regarding the competence and motivation of each of the components of the vaccines value chain (v.gr., the pharmaceutical business, investigators, and health professionals), i.e., the elite, in populist terms [22].

At the individual level, thoughts and feelings can influence getting vaccinated [23]. Vaccine attitudes lie on a spectrum between the extremes that represent those people that have fixed anti-vaccination or pro-vaccination views [24]. Active refusal is not the main concern for the 20 vaccines that are normally included in most of the high-income countries’ routine vaccination schedules [25,26]. Delaying or spreading out vaccination is a major concern [23].

For understanding the context in which individual vaccination decisions operate, we first have to know how the actors perceive the risk involved and how it influences their behaviour. Considering the negative impact that vaccine fear has on people’s behaviour, public health experts have prioritized confidence in vaccines as a key driver in vaccination [27,28]. A second key component of vaccine confidence relates to trust in the social institutional system in which vaccination occurs [29]. Citizens usually receive vaccination from a healthcare professional (physician, nurse, or pharmacist), so that trust in them seems to be a very important relationship to consider. Nevertheless, several studies showed that variation in trust of healthcare providers does not explain variation in vaccination coverage [30,31]. The influence of trust in vaccination behaviour must consider other social institutions that could exert more impact in the vaccination decision-process. Among them, we found the information media, the political parties, the regional or local public authorities, the national government, the national Parliament, and the European Union [32,33,34,35].

Scales for measuring attitudes toward vaccines have been usually developed for specific infectious diseases [23]. When measuring confidence toward vaccines in general, there is evidence that a simplified scale that only considers the perceived benefits of vaccines performs as well as the entire scale [29]. As the goal of our research is to develop a parsimonious invariant measurement model for testing the relationship between vaccine hesitancy and populism in general terms, only two drivers would be considered: the usefulness of being vaccinated (hereinafter referred as USELESS because it is reverse coded) and trust in the institutions that form the vaccine environment (hereinafter referred as DISTRUST because it is reverse coded).

**Figure 1:**
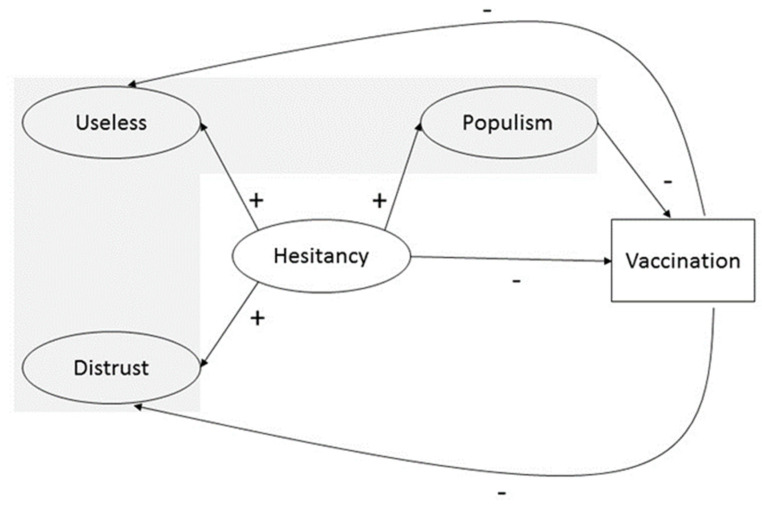
Conceptual Model. The grey-shaded area represents the measurement model composed of three latent variables (Useless, Distrust and Populism). The full conceptual model added a second order latent variable that constituted vaccine hesitancy (Hesitancy) and an observed variable that exhibited vaccine uptake (Vaccination). Arrows and signs represent the hypothesized relationships among the concepts.

**Figure 2:**
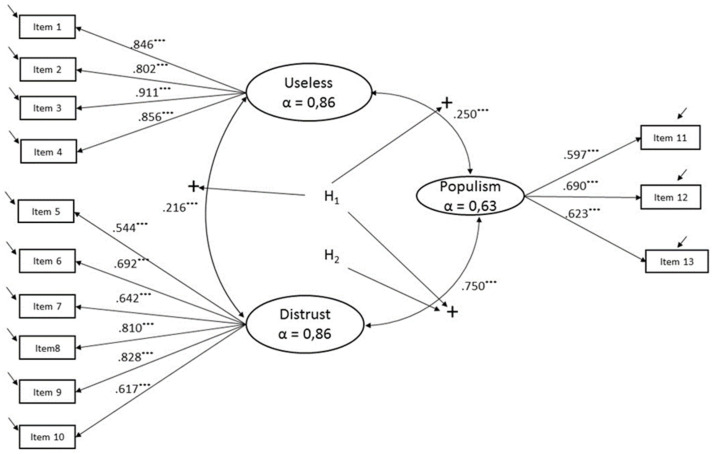
Measurement Model. The latent variables proposed in the model (circled) and the signs hypothesized in their relationships are depicted in the center of the figure. Each latent variable has its associated Conbrach’s alpha (α). All of the path loads from latent variables to items are in standardized terms. *** represents *p*-values significant at the 1% level of significance.

Hence, our measurement model was composed of three variables: two (USELESS and DISTRUST) that comprised vaccine-hesitancy (hereinafter referred as HESITANCY), and one that represented the politic populism (hereinafter referred as POPULISM) (see the grey-shaded area in Figure 1). Once the measurement model proposed is stated as invariant in all countries under study, we expected that the country means for each of the latent variables would show differences among countries. We hypothesized, following Kennedy [2], a highly positive significant association between vaccine hesitancy and political populism. We expected that the positive association between the variables would be even higher in the case of the DISTRUST-POPULISM because, as previously explained, distrust in institutions is one of the main key drivers of political populism and the nexus with vaccine hesitancy (see Figure 2).

**Hypothesis** **1** **(H1).** *USELESS, DISTRUST, and POPULISM are positively associated*.

**Hypothesis** **2** **(H2).** *DISTRUST and POPULISM have the highest positive association*.

**Hypothesis** **3** **(H3).** *POPULISM and VACCINE HESITANCY have a positive significant association across countries*.

## 3. Materials and Methods

### 3.1. Sample

The data stem from the EUROBAROMETER survey 91.2 that was carried out between the 15th and the 29th of March 2019 by the company Kantar Public, at the request of the European Commission [36]. The dataset was accessed through GESIS (Leibniz-Institute für Sozialwissenschaften, University of Cologne, Germany) at https://www.gesis.org/ (accessed on 16 October 2021). The EUROBAROMETER is part of wave 91.2 and covers the population of the respective nationalities of the European Union member states, residents in each of the member states, and aged 15 years and over. In these countries, the survey covers the national population of citizens of the respective nationalities and the population of citizens of all of the European Union member states that are resident in those countries and have a sufficient command of one of the respective national language(s) to answer the questionnaire. The basic sample design applied in all states is a multi-stage random one, totalling 27,524 respondents (see Table A2 in the Appendix A).

### 3.2. Instrumentation

The survey measured vaccine usefulness (Cronbach’s Alpha = 0.86) using four items. Each item—“To what extent do you agree or disagree with the following statements …” Item 1. “It is important for everybody to have routine vaccinations”, Item 2. “Not getting vaccinated can lead to serious health issues”, Item 3. “Vaccines are important not only to protect yourself but also others”, Item 4. “Vaccination of other people is important to protect those that cannot be vaccinated …”—was measured on a four-point scale ranging from 1 “Totally agree” to 4 “Totally disagree”. As the scale was reversed, we named the latent variable obtained from these indicators as USELESS. We included six items for measuring trust (Cronbach’s alpha = 0.77)—“… how much trust you have in certain media and institutions…” Item 5. “The media”, Item 6. “Political parties”, Item 7. “Regional or local public authorities”, Item 8. “The national government”, Item 9. “The national parliament”, Item 10. “The European Union”. Respondents expressed their agreement with these statements on a two-item scale from 1. “Totally agree” to 2. “Totally disagree”. As the scale was also reversed, we choose to name the resulting latent variable as DISTRUST. Finally, the survey asked the interviewees three questions that formed POPULISM (Cronbach’s alpha = 0.63). The first one (Item 11), measured on a four-point scale from 1. “Totally agree” to 4. “Totally disagree”, was: “The interests of people like you are well taken into account by the political system in (OUR COUNTRY)”. The second one (Item 12), measured on a four-point scale from 1. “Totally agree” to 4. “Totally disagree”, questioned “On the whole, are you very satisfied, fairly satisfied, not very satisfied, or not at all satisfied with the way democracy works in (OUR COUNTRY)?” The third one (Item 13), asked the participants “At the present time, would you say that, in general, things are going in the right direction or in the wrong direction, in (OUR COUNTRY)?” We recoded this indicator in 1. “Things are going in the right direction”, 2. “Neither the one nor the other (SPONTANEOUS), 3, “Things are going in the wrong direction”. The measurement model proposed could be seen in Figure 2.

### 3.3. Statistical Analysis

From a methodological perspective, as we compared data from 28 different countries to check how the vaccine hesitancy’s drivers (USELESS and DISTRUST) were related to the populist political ideology, we first assessed that the answers were comparable and that the proposed measurement model applies in all countries (measurement invariance, MI) [37]. Following the analysis made by Muthén and Asparouhov [38], from the different methods available to evaluate MI, alignment should be the chosen one. This election was made based on the following reasons. First, the alignment method works very well with a small number of indicators. Second, the number of groups is less than 30 (28 countries in our study). Third, for accomplishing the goals of our research it is very important to know which countries contribute to non-invariance. The alignment method conveniently provides this information when compared with the other methodological alternatives (approximate Bayesian invariance or multilevel random intercepts/random slopes approach). Finally, considering that our observed indicators are measured in Likert scales, the alignment method is better because it is nonparametric, allowing any kind of measurement parameter distribution. 

For evaluating the MI of the proposed model, the first step was to check if there was an identity of parameters among countries through a Multi-Group Confirmatory Factor Analysis (MGCFA) [39]. MGCFA helped in answering to what degree the proposed model was invariant/equivalent across European countries. Three (increasingly restrictive) types of identity were assessed: configural, metric, and scalar invariance. Configural invariance requires that the same items load on the same factor(s) in all countries [40]. If the items are associated with the latent factor(s) as expected, then configural invariance holds [41]. A scale has metric invariance when the magnitudes of the relationships between items (factor loadings) and latent factors are equivalent among countries. If the model fit worsens in comparison with the configural one, we would proceed with examining partial invariance [42]. If the metric invariance is supported, we proceed to test scalar invariance. This occurs when, additionally to the previous requisites, intercepts are equivalent among countries [41]. Scalar invariance is established when the fit of the model, with factors loadings and indicators, and intercepts constrained to be equal, does not worsen from the fit of the metric model [43]. One of the main goals of our paper intended to establish meaningful comparisons among the three factors’ (useless, distrust, and populism) means across the European countries. Hence, we needed scalar invariance.

The notations of these three levels of invariance for a particular item of each factor are (Equations (1)–(3)):

Configural:

(1) $$y_{ij}=v_{j}+\lambda_{j}\;f_{ij}+\varepsilon_{ij}\;E\left(f_{i}\right)=\alpha_{j}=0,\;V\left(f_{i}\right)=\psi_{j}=1$$

Metric:

(2) $$y_{ij}=v_{j}+\lambda f_{ij}+\varepsilon_{ij}\;E\left(f_{i}\right)=\alpha_{j}=0,\;V\left(f_{i}\right)=\psi_{j}=1$$

Scalar:

(3) $$y_{ij}=v+\lambda \;f_{ij}+\varepsilon_{ij}\;E\left(f_{i}\right)=\alpha_{j},\;V\left(f_{i}\right)=\psi_{j}$$

where v is a measurement intercept, λ is a factor loading, f is a factor with mean α and variance ψ, and ε is a residual with mean zero and variance *θ* uncorrelated with f. 

As we already mentioned in the first paragraph of this section, among the different alternatives available when invariance did not meet, we chose the alignment method. Following the reasoning made by Asparouhov and Muthén [44] and its implementation in the software MPLUS 8.6 ( Muthén & Muthén, Los Angeles, CA, USA) [45], we began estimating the configural model letting loadings and intercepts free across countries, factor means fixed at 0 in all countries, and factor variances fixed at 1 in all countries. Then, we proceeded with the alignment optimization freeing the factor means and variances and choosing their values to minimize the total amount of non-invariance [44,46]. 

## 4. Results

As a first step, we checked if the hypothesized measurement model (see Figure 2) fits well for each European country under study (separately calculated). In order to evaluate whether measurement invariance was supported, we first performed chi-square difference tests. Chi-square tests are overly sensitive when, as in our study, sample sizes are large and when the data is not normally distributed. In these cases, even substantially irrelevant differences can turn up as statistically significant [47]. This limited the usefulness of this index in our analysis. Additionally, we considered three global fit measures: the comparative fit index (CFI), the root mean square error of approximation (RMSEA), and the standardized root mean residual (SRMR). We considered models with a CFI value higher than 0.90, and RMSEA and SRMR values lower than 0.08 as acceptable [48,49,50]. Fit indices for all European countries met the generally accepted criteria (see Appendix A, Table A3).

In step two, we tested the configural invariance. This meant that we checked that each latent variable in the measurement model had the same set of indicators in each country, the model fits the data well in each country, and all factor loadings are substantial. Fit indices met the aforementioned criteria: χ^2^(2828) = 7642.839, *p* = 0.000; RMSEA = 0.042; CFI = 0.966; SRMR = 0.052 (see Table 1). 

In step three, the fit of the model with all factor loadings constrained across the 28 European countries (metric model) was compared to the fit of the configural model. If the model fit did not significantly decrease after imposing these restrictions, the more restrictive model (metric model) could be accepted. As previously explained, the change in χ^2^ was statistically significant [χ^2^(3179) = 11507.306, *p* = 0.000] (see Table 1), but was not very useful because of the test’s high sensitivity to the large sample used. For that reason, we followed the suggestions made by Chen [51] who advised considering the differences in other model fit statistics (CFI, RMSEA, and SRMR). With N > 300, differences between configural and metric models are considered relevant when the change in CFI is larger than 0.010, accompanied by a change in RMSEA larger than 0.015, or a change in SRMR larger than 0.030. The variation in RMSEA and SRMR held with the maximum 0.01 recommended, but the 0.03 variation in CFI exceeded it. 

The metric and scalar models showed many large modification indices. This implied that for reaching a final good scalar model with acceptable fit indices, too many modifications were needed. Furthermore, the final result could lead us to a wrong model.

As we already mentioned in the methodological section, for overcoming the non-invariance we applied the alignment method. The advantage of the alignment method is that metric and scalar invariance are not required for comparing factor means among countries because it makes them comparable while minimizing measurement non-invariance [44]. We ran the alignment procedure using the software MPLUS 8.6 [45] with the country with the smallest factors mean (the Netherlands in our case) as the reference group [52]. Results reached an overall non-invariance of 21.5% ( see Appendix A, Table A4). This result met the recommended rule of thumb (lower than 25% [53]) and could be considered acceptable. Additionally, we also summarized in Table 2 the fitting functions of both the factor loading and intercept for each item considered in the latent variables of our model. R2 values in this table corroborated these results.

For supporting the acceptance of the alignment result, we also performed Monte Carlo simulations based on the alignment parameter estimates for testing that factor means were well estimated so that countries’ comparisons could be made. In doing so, we used the number of items, the number of countries, the degree of measurement non-invariance, the country-varying factor means and variances, and the sample sizes in the countries. We carried out the analysis using MPLUS 8.6 software [45] performing 1000 replications. In order to be trustworthy, a near-perfect correlation was required—at least 0.98—between the estimated factor means and the generated Monte Carlo ones [38]. In our case, the correlations were USELESS = 1.000, DISTRUST = 0.999, and POPULISM = 0.998, suggesting excellent alignment despite non-invariance.

**Figure 3:**
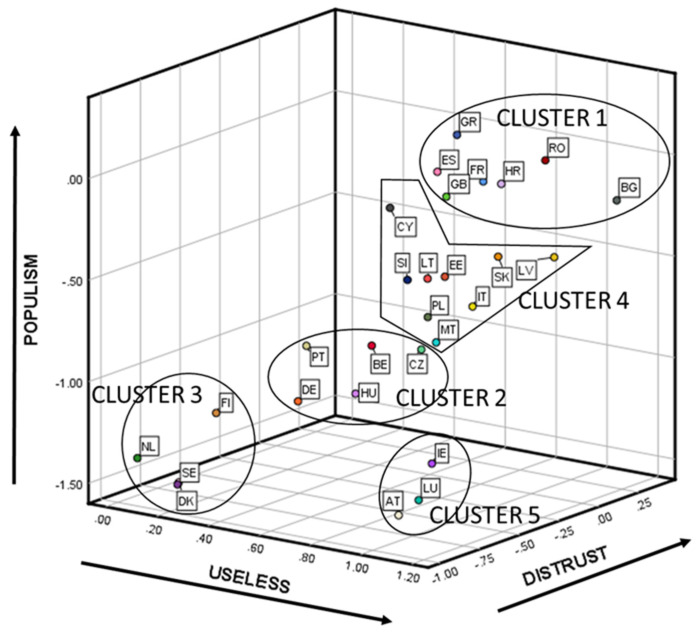
Factor Means for USELESS, DISTRUST, and POPULISM in 28 Countries. Alignment Method. Country codes and number of respondents per country are available in Table A2, and numerical results in Table A5, both in Appendix A.

All of the previous results allowed us to feel confident in the reliability of the latent mean estimates and their comparisons across 28 countries. For better interpreting the results, the factor means for each of the 28 countries can be seen in Figure 3 (country codes and number of respondents per country are available in Table A2, and numerical results in Table A5, both in Appendix A).

For simplifying the interpretation of the results in a data-analytic way, we clustered the factor means among countries concerning their similarities. Using the factor means for each of the 28 countries obtained in the previous step, we proceeded to analyze the different country segments that existed. For choosing the best clustering method we used the package “clValid” from R [54]. Hierarchical methods performed better than K-Means and PAM for the three internal measures of clustering validation used (Connectivity, Dunn, and Silhouette). Considering the compactness, separation, connectivity, and interpretability, the five-cluster solution performed the best. Figure 3 visually depicts the results of the hierarchical clustering approach (using the squared Euclidean distance and the Ward method). For testing whether the country segments found were differentiable, we calculated segments’ means differences by applying an ANOVA-Tukey HSD analysis (see Table 3). From these results, we concluded that the country segments’ mean differences were statistically significant for DISTRUST—except when comparing cluster 5 and cluster 2—and POPULISM—except when comparing clusters 5 and 3. USELESS only had statistically significant mean differences when comparing cluster 3 with any of the others. 

The characteristics of each of the found segments were:

- Cluster 1. The countries that belonged to this cluster were France, United Kingdom, Spain, Greece, Romania, Bulgaria, and Croatia. They showed the highest mean in POPULISM, DISTRUST, and USELESS—in this latter case only surpassed by cluster 5, but not statistically significant.
- Cluster 2. The countries that formed this cluster were Germany, Portugal, Belgium, Hungary, and the Czech Republic. They presented a medium position in POPULISM—higher than clusters 3 and 5 and lower than clusters 1 and 4—and a medium-low position in DISTRUST—higher than cluster 3, lower than clusters 1 and 4 and not statistically significantly different from cluster 5. USELESS did not have any statistically significant difference with any other cluster.
- Cluster 3. The countries that pertained to this cluster were The Netherlands, Finland, Sweden, and Denmark. They displayed one of the two lowest positions in POPULISM and DISTRUST—shared with cluster 5. USELESS, especially useful for marking the difference between this cluster and all the rest, presented the lowest position.
- Cluster 4. The countries related to this cluster were Italy, Poland, Slovakia, Estonia, Latvia, Lithuania, Slovenia, Cyprus, and Malta. POPULISM and DISTRUST exhibited a medium-high level—higher than clusters 2, 3, and 5, and lower than cluster 1. The only statistically significant difference found for USELESS was that previously commented with cluster 3.
- Cluster 5. The countries connected to this cluster were Austria, Luxemburg, and Ireland. As already mentioned when describing cluster 3, this cluster had the lowest positions in POPULISM and DISTRUST, tied with cluster 3, but not in USELESS, which was in line with the mean of clusters 1, 2, and 4.

## 5. Discussion and Conclusions

As a compulsory analytic prerequisite to making comparisons when data from large-scale surveys in several countries are used, we chose the alignment method that allowed us to check the invariance of the proposed measurement model. We reached a parsimonious invariant measurement model for studying the relationships between vaccine hesitancy and political populism in EUR 27 + UK.

From the two latent variables studied that we used for measuring vaccine hesitancy, DISTRUST had the strongest relationship with POPULISM (see Figure 2) while USELESS was only statistically significant for differentiating cluster 3 from all the others (see Appendix A, Table A4). Hence, Hypotheses 1 and 2 holds.

Several European countries felt that the vaccine’s usefulness to fight against infectious diseases was not very high. Only the countries that belong to Cluster 3 (Denmark, The Netherlands, Sweden, and Finland) showed sound confidence in vaccines. For the rest of the country clusters, the perception of vaccines’ uselessness could be explained by different reasons. Some European countries that were exposed to Soviet communism (Bulgaria, Latvia, Romania, and Slovakia) depicted higher rates of vaccine USELESS ( see Appendix A, Table A5). Several investigations have found that weak trust in government, medical personal, and medical advice from doctors explained these cases [55,56,57]. For some other eastern European countries that were also under the influence of Soviet communism (mainly the Czech Republic, Poland, and Hungary), the higher usefulness that they portrayed in vaccines could be explained by their experience developing vaccines since the 1950s and the role that they played in the propaganda campaigns claiming credit for their superiority [58]. The rest of the ex-communist states felt, in these moments, the authoritarianism inherent in communist states when imposing vaccination trials and uptakes, which generated anti-vaccine sentiments that have remained ever since. Furthermore, not all the countries with low levels of DISTRUST and POPULISM believed that vaccines were useful for fighting against infectious diseases. As we saw, all the countries that belonged to cluster 5 (Austria, Luxembourg, and Ireland) felt that vaccines were useless. From a social marketing perspective, the results obtained for USELESS suggested that, except for countries that belonged to Cluster 3, marketing actions needed to be implemented to reinforce the association of vaccines as a proven and successful solution to fight against infectious diseases.

Distrust in institutions was the main underlying driver that was associated with both vaccine hesitancy and political populism (see Figure 2). We also saw (Figure 3) that a positive relationship existed between the two variables that formed vaccine hesitancy (USELESS and DISTRUST) and POPULISM. Hence, Hypothesis 3 also holds. 

The challenge that the world seems to be facing is trustdemic: falling public trust is all around the world [59,60,61]. This could explain how vaccine-hesitancy is increasing worldwide while having highly effective vaccines. Trusted institutions are the grease in the social machine. As Khanna [62] said, “When citizens lack trust, they are less likely to comply with laws and regulations, pay taxes, tolerate different viewpoints or ways of life, contribute to economic vitality, resist the appeals of demagogues, or support their neighbours. Without trust, societies are at risk of chaos and conflict.”

Populist political parties know that vaccines are fertile ground for instilling doubt and trying to gain from polarized debates [63]. The main strategy of populists is to polarize pro and con views on vaccines, joining them with any other sentiments (anti-chemical, anti-science, anti-migration, anti-abort, anti-government, etc.) [64]. Results from this strategy were clear: our measurement model portrayed a strong positive relationship among DISTRUST and POPULISM in all the European countries.

Vaccine-hesitancy is also very adequate for the populism strategy due to the “small pockets” issue. They do not need to convince the whole population to follow a vaccine-hesitant attitude. “Herd immunity”, the level at which immunization coverage has to be maintained in order to be effective, depends on the vaccine but it typically ranges between 80% and 90% of the population [23,65]. Hence, a small number of vaccine-hesitants can have enormous adverse effects on herd immunity and epidemic spread. Even more, if we consider that this low percentage that is leftover to attain “herd immunity” has to be reserved for people with compromised immune systems or for those that are too young, for example, neonates.

We also must bear in mind that to be vaccine-hesitant does not necessarily imply that one does not take any vaccines. Willingness to be vaccinated lies in a continuum ranging from those who accept all vaccines without any doubt to those who reject all without any doubt. It is complex and context-specific, varies across time, place, and vaccines [65,66]. Therefore, from the willingness to be vaccinated to the uptake there is still a myriad of factors that could influence the final decision and that belong to the demand side of vaccination [8,67]. One of them could be how populism affects vaccination uptake. It has not been studied in this paper. The mediation role of populism on vaccine uptake, depicted in Figure 1, is part of an ongoing research study that has obtained promising preliminary results.

From the results, there are several implications for management. First, sixteen out of twenty-eight European countries under study showed high rates of distrust in institutions and populism and medium-low levels of confidence in vaccines for fighting against infectious diseases. They are all the countries that belong to Cluster 1 and Cluster 4. Populist organizations likely take advantage of this situation for generating social instability based on vaccine-hesitancy. Second, having low levels of distrust and populism does not mean living without problems. Countries that compose Cluster 3 have the highest perception that vaccines are not useful. Hence, vaccine hesitancy could be high and populist organizations could profit from this situation. Third, countries that constitute cluster 3 are at the medium of the spectrum. The two countries that belonged to the former Soviet communism area of influence (Hungary and Czech Republic) are proud of the role played during the Cold War when developing vaccines. Nevertheless, the economic, political, and economic situation in these countries could make them follow the same path as Poland (also proud of their vaccine experience in the past, but with higher levels of distrust and populism). Germany shares a similar problem: the *landers* that were in the Soviet communism area of influence have higher degrees of vaccine hesitancy and populist organizations are making use of it [68]. Fourth, countries included in Cluster 3 portray the best position represented by the low rates of the three drivers. Nevertheless, the “small pockets” concern could be a problem in these countries due to the existence of global vaccine-hesitant segments [69].

Social marketing must be targeted for convincing vaccine-hesitants that to be vaccinated is a societal health strategy and not just a question about individual rights. Proactive steps must be implemented for restoring the trust in scientists that develop vaccines, governments, institutions, and businesses. A lifetime acceptance of vaccine programs, orchestrated through a relationship marketing campaign, could be the basis of a trust chain for better resisting the negative economic and social outcomes derived from the association that this paper shows between populism and vaccine-hesitancy.

## 6. Limitations

The present study has some limitations. The clearest one is the use of the Eurobarometer’s predefined items. Nevertheless, the benefit of the large-scale surveys carried out by well-known public international organizations is the high quality of the data obtained through a standardized sampling procedure. It was intended by the authors to fill the gap that existed in the vaccine-hesitancy literature for testing the invariance of the measurement items used when several countries were present. For this purpose, Eurobarometer’s data fit perfectly.

Further research is necessary to quantify how populism mediates the final vaccination uptake decision. The limitation of using MGFA with alignment for validating the proposed measurement model could be relaxed—including covariates and using full structural equation modeling—when testing the mediation model proposed in Figure 1.

Finally, data used for the analysis were gathered between the 15th and the 29th of March 2019, before the COVID-19 pandemic. New more recent data are needed for reinforcing the proposed relationship between vaccine hesitancy and populism.

## Figures and Tables

**Table 1:** MGCFA Model Fit. Configural, Metric and Scalar Invariance Analysis.

Test Results	Configural	Metric	Scalar
χ^2^	7642.839	11507.306	17318.669
χ^2^ df	2828	3179	3530
χ^2^ *p*-Value	0.000	0.000	0.000
RMSEA	0.042	0.052	0.063
ΔRMSEA		0.01	0.011
CFI	0.966	0.941	0.903
ΔCFI		−0.025	−0.038
SRMR	0.052	0.062	0.072
ΔSRMR		0.01	0.01

Note: χ^2^ = chi-square, χ^2^ df = chi-square degrees of freedom, χ^2^ *p*-value = chi-square *p*-value, RMSEA = Root Mean Square of Approximation, CFI = Comparative Fit Index, SRMR = Standardized Root Mean Residual, ΔRMSEA = difference in RMSEA from the previous step, ΔCFI = difference in CFI from the previous step, ΔSRMR = difference in SRMR from the previous step.

**Table 2:** Alignment Fit Statistics.

	Factor Loadings		Intercepts		Factor Loadings + Intercepts
Item Code	Fit Function Contribution	R2	Fit Function Contribution	R2	Total Contribution
Useless					
Item 1	−136.881	0.878	−163.207	0.814	−300.088
Item 2	−134.464	0.898	−152.257	0.902	−286.721
Item 3	−131.675	0.943	−127.993	0.969	−259.668
Item 4	−130.332	0.943	−133.887	0.940	−264.220
Distrust					
Item 5	−164.872	0.249	−242.401	0.509	−407.273
Item 6	−187.521	0.452	−197.492	0.825	−385.013
Item 7	−165.045	0.346	−183.488	0.789	−348.532
Item 8	−145.813	0.649	−162.651	0.815	−308.465
Item 9	−150.295	0.663	−173.275	0.882	−323.570
Item 10	−159.713	0.103	−217.085	0.000	−376.798
Populism					
Item 11	−143.368	0.624	−187.273	0.727	−330.641
Item 12	−149.007	0.763	−201.916	0.869	−350.923
Item 13	−185.306	0.557	−226.041	0.737	−411.347

Note: The R2 value gives the parameter variation across groups in the configural model that is explained by variation in the factor mean and factor variance across groups. A value close to 1 implies a high degree of invariance and a value close to 0 implies a low degree of invariance (Asparouhov and Muthén, 2014, p. 6 [44]).

**Table 3:** ANOVA (Tukey HSD). Country-Segment’s Mean Differences.

Country-Segments	Useless	*p*	Distrust	*p*	Populism	*p*
2-1	−0.26	*n.s.*	−0.52	***	−0.86	***
3-1	−0.64	***	−0.99	***	−1.27	***
4-1	−0.04	*n.s.*	−0.23	*	−0.46	***
5-1	0.08	*n.s.*	−0.64	***	−1.37	***
3-2	−0.38	*	−0.48	***	−0.41	***
4-2	0.22	*n.s.*	0.28	*	0.4	***
5-2	0.34	*n.s.*	−0.12	*n.s.*	−0.51	***
4-3	0.6	***	0.76	***	0.81	***
5-3	0.72	***	0.35	*n.s.*	−0.09	*n.s.*
5-4	0.12	*n.s.*	−0.41	**	−0.91	***

Note: Country-Segments indicate the segment code number as depicted in Figure 3. Level of significance: * *p* ≤ 0.05, ** *p* ≤ 0.01, *** *p* ≤ 0.001, *n.s.* not significant.

## Data Availability

Publicly available datasets were analyzed in this study. This data can be found through GESIS (University of Cologne, Germany) at https://www.gesis.org/en/eurobarometer-data-service/search-data-access/data-access (accessed on 16 October 2021).

## Appendix A

**Table A1:** Search Strategy in Web of Science in order to know how many vaccine hesitancy publications used measurement invariance analysis.

	Search Terms	Hits
#1	TI=( “vaccin* hesitan*” OR “hesitan* to vaccine*” OR “vaccin* refusal” OR “refusal to vaccine*” OR “vaccin* opposition” OR “opposit* to vaccin*” OR “antivacc* group*” OR “antivax” OR autovaccination OR “object* to vaccin*” OR “resilience to vaccin*” OR “debate against vaccin*” OR “vaccin** compliance” OR “vaccin** adherence” OR “resist* to vaccin*” OR “incomplete vaccin*” OR “misinformation about vaccine*” OR “vaccin* criticism*” OR “delaying vaccin*” OR “anxiety from vaccin*” OR “criticism to vaccin*” OR “barrier* to vaccin*” OR “lack of intent to vaccin*” OR “poor completion of vaccin*” OR “compulsory vaccin*” OR “negative perception about vaccin*” OR “ negative attitudes vaccin*” OR “engagement in vaccin*” OR “choice to vaccin*” OR “awareness about vaccin*” OR “knowledge about vaccin*” OR “behavi* toward vaccin*” OR “poor vaccin* uptake” OR “vaccin* uptake rate” OR “doubts about vaccin*” OR “acceptance of vaccin*” OR “acceptability of vaccin*” OR “contravers* about vaccin*” OR “religious exemption vaccine*” OR “fear from vaccin*” OR “belief in vaccin*” OR “mandatory vaccin*” OR “compulsory vaccin*” OR “willingness to accept vaccin*” OR “parental control of child* vaccin*” OR “willingness to vaccine*” OR “willingness to accept vaccin*”)	1663
#2	AB=(“vaccin* hesitan*” OR “hesitan* to vaccine*” OR “vaccin* refusal” OR “refusal to vaccine*” OR “vaccin* opposition” OR “opposit* to vaccin*” OR “antivacc* group*” OR “antivax” OR autovaccination OR “object* to vaccin*” OR “resilience to vaccin*” OR “debate against vaccin*” OR “vaccin** compliance” OR “vaccin** adherence” OR “resist* to vaccin*” OR “incomplete vaccin*” OR “misinformation about vaccine*” OR “vaccin* criticism*” OR “delaying vaccin*” OR “anxiety from vaccin*” OR “criticism to vaccin*” OR “barrier* to vaccin*” OR “lack of intent to vaccin*” OR “poor completion of vaccin*” OR “compulsory vaccin*” OR “negative perception about vaccin*” OR “ negative attitudes vaccin*” OR “engagement in vaccin*” OR “choice to vaccin*” OR “awareness about vaccin*” OR “knowledge about vaccin*” OR “behavi* toward vaccin*” OR “poor vaccin* uptake” OR “vaccin* uptake rate” OR “doubts about vaccin*” OR “acceptance of vaccin*” OR “acceptability of vaccin*” OR “contravers* about vaccin*” OR “religious exemption vaccine*” OR “fear from vaccin*” OR “belief in vaccin*” OR “mandatory vaccin*” OR “compulsory vaccin*” OR “willingness to accept vaccin*” OR “parental control of child* vaccin*” OR “willingness to vaccine*” OR “willingness to accept vaccin*”)	4333
#3	(#1) OR #2	5225
#4	TI = (“measurement invariance” OR “multigroup invariance” OR “Multi-group confirmatory factor analysis” OR “factorial invariance” OR “measurement equivalence” OR MGCFA )	2267
#5	AB = (“measurement invariance” OR “multigroup invariance” OR “Multi-group confirmatory factor analysis” OR “factorial invariance” OR “measurement equivalence” OR MGCFA)	5614
#6	(#4) OR #5	6174
#7	TI = (“configural” AND “metric” AND “scalar”)	4
#8	AB = (“configural” AND “metric” AND “scalar”)	616
#9	(#7) OR #8	618
#10	(#6) OR #9	6337
#11	(#3) AND #10	2

Source: Adapted from Sweileh [70].

The query was made from 1900 to July, the 22nd 2021 in the Web of Science Core Collection, Current Contents Connect, Derwent Innovation Index, KCI- Korean Journal Database, MEDLINE, Russian Science Citation Index, and SciELO Citation Index. Search strategy consisted in searching for the selected key terms in the Web of Science’s “Title” and “Abstract” fields. Some changes were made to the original key terms used by Sweileh (2020) because of the appearance of false positives when validating the final results. No language restriction was made.

**Table A2:** Reference codes, sample size by country, and total population 15+.

Country	Country Code	Country Code Number	Number of Interviews	Population 15+
France	FR	1	1013	54,097,255
Belgium	BE	2	1041	9,693,779
The Netherlands	NL	3	1017	13,979,215
Germany	DE	4	1507	70,160,634
Italy	IT	5	1021	52,334,536
Luxemburg	LU	6	512	457,127
Denmark	DK	7	1017	4,838,729
Ireland	IE	8	1078	3,592,162
United Kingdom	UK	9	1021	52,651,777
Greece	GR	10	1014	9,937,810
Spain	ES	11	1014	39,445,245
Portugal	PT	12	1013	8,480,126
Finland	FI	13	1000	4,747,810
Sweden	SE	14	1021	7,998,763
Austria	AT	15	1006	7,554,711
Republic of Cyprus	CY	16	505	741,308
Czech Republic	CZ	17	1068	9,238,431
Estonia	EE	18	1005	1,160,064
Hungary	HU	19	1030	8,781,161
Latvia	LV	20	1012	1,707,082
Lithuania	LT	21	1004	2,513,384
Malta	MT	22	497	364,171
Poland	PL	23	1011	33,444,171
Slovakia	SK	24	1020	4,586,024
Slovenia	SI	25	1016	1,760,032
Bulgaria	BG	26	1026	6,537,535
Romania	RO	27	1025	16,852,701
Croatia	HR	28	1010	3,796,476
TOTAL			27,524	431,452,219

Source: Eurobarometer 91.2. European Commission [36].

**Table A3:** Fit Indices of the Measurement Model in Each Country.

Country	χ^2^	χ^2^ df	χ^2^ *p*	RMSEA	CFI	SRMR
France	217.747	62	0.000	0.050	0.955	0.035
Belgium	157.106	62	0.000	0.038	0.972	0.033
The Netherlands	140.942	62	0.000	0.035	0.975	0.030
Germany	252.921	62	0.000	0.045	0.967	0.031
Italy	261.537	62	0.000	0.056	0.958	0.037
Luxembourg	128.510	62	0.000	0.050	0.960	0.040
Denmark	156.490	62	0.000	0.040	0.970	0.030
Ireland	152.800	62	0.000	0.040	0.980	0.030
United Kingdom	213.690	62	0.000	0.050	0.960	0.030
Greece	261.640	62	0.000	0.060	0.940	0.030
Spain	183.460	62	0.000	0.040	0.960	0.030
Portugal	204.470	62	0.000	0.050	0.970	0.030
Finland	198.010	62	0.000	0.050	0.960	0.040
Sweden	112.670	62	0.000	0.030	0.980	0.030
Austria	326.950	62	0.000	0.060	0.910	0.050
Cyprus (Republic)	113.250	62	0.000	0.040	0.970	0.040
Czech Republic	215.590	62	0.000	0.050	0.960	0.040
Estonia	203.050	62	0.000	0.050	0.960	0.030
Hungary	408.160	62	0.000	0.070	0.940	0.040
Latvia	227.280	62	0.000	0.050	0.950	0.040
Lithuania	157.190	62	0.000	0.040	0.970	0.030
Malta	271.480	62	0.000	0.080	0.920	0.050
Poland	364.660	62	0.000	0.070	0.900	0.060
Slovakia	523.750	62	0.000	0.080	0.910	0.050
Slovenia	153.170	62	0.000	0.040	0.980	0.030
Bulgaria	285.100	62	0.000	0.060	0.950	0.040
Romania	318.880	62	0.000	0.060	0.930	0.050
Croatia	304.180	62	0.000	0.060	0.950	0.040

Note: χ^2^ = chi-square, χ^2^ df = chi-square degrees of freedom, χ^2^ *p* = chi-square *p*-value, RMSEA = Root Mean Square of Approximation, CFI = Comparative Fit Index, SRMR = Standardized Root Mean Residual.

**Table A4:** Alignment results. Approximate measurement (non) invariance for intercepts and loadings, 28 countries.

Variable/Item	Intercept	Loadings
Useless		
Item 1	(1) 2 3 4 (5) (6) 7 (8) (9) 10 11 12 13 (14) 15 16 (17) 18 19 20 21 22 (23) 24 25 26 27 28	1 2 3 4 5 6 7 8 9 10 11 12 13 14 15 16 17 18 19 20 (21) 22 (23) 24 25 26 27 28
Item 2	1 2 (3) 4 5 6 (7) (8) 9 10 11 (12) (13) 14 15 16 (17) 18 (19) 20 21 22 (23) 24 25 26 (27) 28	(1) 2 3 4 5 6 7 8 9 10 11 12 (13) 14 15 16 17 18 19 20 21 22 23 24 25 26 27 28
Item 3	1 2 3 4 5 6 7 8 9 10 11 (12) 13 14 15 16 17 18 19 20 21 22 23 24 25 26 27 28	1 2 3 4 5 6 (7) 8 9 10 11 12 (13) 14 15 16 17 18 19 20 21 22 23 24 25 26 27 28
Item 4	1 2 (3) 4 5 6 7 8 9 10 11 12 13 14 15 16 17 18 19 20 21 22 (23) 24 25 26 27 28	1 2 3 4 5 6 7 8 9 10 11 12 13 (14) 15 16 17 18 19 20 21 22 23 24 25 26 (27) 28
Distrust		
Item 5	1 2 (3) 4 5 6 (7) 8 (9) 10 11 (12) (13) 14 15 16 (17) (18) (19) (20) (21) (22) 23 24 25 (26) (27) 28	1 2 3 4 5 6 7 8 9 10 11 (12) 13 14 15 16 17 18 19 20 21 22 23 24 25 26 27 28
Item 6	1 2 3 4 (5) 6 7 8 9 10 11 12 13 14 15 16 17 18 19 (20) 21 22 23 24 25 26 27 (28)	(1) 2 (3) 4 5 6 7 8 9 (10) (11) (12) 13 14 15 (16) 17 18 19 (20) 21 22 23 24 (25) 26 27 (28)
Item 7	(1) 2 3 (4) (5) 6 (7) (8) 9 (10) (11) (12) 13 14 15 (16) 17 18 19 20 21 (22) (23) (24) (25) (26) (27) (28)	1 2 3 (4) (5) 6 7 8 9 (10) 11 (12) 13 14 15 16 17 18 19 20 21 22 23 24 (25) 26 27 (28)
Item 8	1 (2) 3 4 5 (6) 7 8 9 10 11 12 13 14 15 16 (17) (18) 19 20 21 (22) 23 24 25 26 (27) (28)	1 2 3 4 5 6 7 8 (9) (10) (11) 12 13 14 15 16 17 18 19 20 21 22 23 (24) 25 26 27 28
Item 9	1 (2) 3 4 5 6 7 (8) 9 (10) (11) (12) (13) (14) 15 16 (17) (18) 19 (20) (21) 22 (23) (24) 25 (26) (27) 28	1 2 3 4 5 6 7 8 9 10 (11) (12) 13 14 15 16 17 18 19 20 21 22 23 24 25 (26) 27 28
Item 10	1 2 (3) (4) 5 6 7 (8) 9 (10) (11) 12 13 14 (15) (16) (17) 18 19 (20) (21) 22 23 (24) (25) (26) (27) (28)	1 2 3 4 5 6 7 8 9 10 11 12 13 14 15 16 17 18 (19) 20 21 (22) (23) 24 25 26 27 28
Populism		
Item 11	1 2 (3) 4 5 6 (7) 8 9 (10) 11 12 (13) 14 15 (16) 17 (18) (19) 20 (21) 22 23 24 25 (26) (27) (28)	1 2 (3) 4 5 6 7 8 9 10 11 12 13 14 15 16 17 18 19 20 21 22 23 24 25 26 27 28
Item 12	1 2 3 (4) (5) 6 (7) 8 9 (10) (11) 12 13 14 15 16 (17) 18 (19) 20 21 (22) (23) 24 25 26 (27) (28)	1 2 3 4 5 6 7 8 9 10 11 12 13 14 15 16 17 18 19 20 21 22 23 24 25 26 27 28
Item 13	1 2 3 4 5 6 7 8 9 10 11 12 13 14 15 16 17 (18) 19 20 (21) (22) 23 24 25 26 27 28	(1) 2 3 4 5 6 7 8 (9) (10) 11 12 13 (14) 15 16 17 (18) 19 20 21 (22) 23 24 25 26 (27) (28)

Note: numbers indicate the country code (see Table A2). The parentheses indicate whether the parameter (intercept or factor loading) is non invariant for that specific group (country code) by variable.

**Table A5:** Factor Mean Comparisons of 28 Countries on USELESS, DISTRUST, AND POPULISM Factors.

Ranking	Country Code	Mean	Countries with Significantly Smaller Factor Mean
*Useless*			
1	26	2.182	27 15 24 5 6 28 1 8 18 21
			22 2 23 19 17 9 10 25 11 16
			4 12 14 13 7 3
2	20	1.973	24 5 6 28 1 8 18 21 22 2
			23 19 17 9 10 25 11 16 4 12
			14 13 7 3
3	27	1.942	24 5 6 28 1 8 18 21 22 2
			23 19 17 9 10 25 11 16 4 12
			14 13 7 3
4	15	1.858	24 5 6 28 1 8 18 21 22 2
			23 19 17 9 10 25 11 16 4 12
			14 13 7 3
5	24	1.602	28 1 8 18 21 22 2 23 19 17
			9 10 25 11 16 4 12 14 13 7
			3
6	5	1.502	21 22 2 23 19 17 9 10 25 11
			16 4 12 14 13 7 3
7	6	1.409	2 23 19 17 9 10 25 11 16 4
			12 14 13 7 3
8	28	1.39	2 23 19 17 9 10 25 11 16 4
			12 14 13 7 3
9	1	1.356	2 23 19 17 9 10 25 11 16 4
			12 14 13 7 3
10	8	1.315	2 23 19 17 9 10 25 11 16 4
			12 14 13 7 3
11	18	1.314	2 23 19 17 9 10 25 11 16 4
			12 14 13 7 3
12	21	1.284	2 19 17 9 10 25 11 16 4 12
			14 13 7 3
13	22	1.27	17 9 10 25 11 16 4 12 14 13
			7 3
14	2	1.108	25 11 16 4 12 14 13 7 3
15	23	1.098	11 16 4 12 14 13 7 3
16	19	1.06	11 16 4 12 14 13 7 3
17	17	1.02	11 16 4 12 14 13 7 3
18	9	1.001	11 16 4 12 14 13 7 3
19	10	0.996	11 16 4 12 14 13 7 3
20	25	0.954	11 4 12 14 13 7 3
21	11	0.784	12 14 13 7 3
22	16	0.756	12 14 13 7 3
23	4	0.74	12 14 13 7 3
24	12	0.524	14 13 7 3
25	14	0.266	3
26	13	0.201	3
27	7	0.193	3
28	3	0	
*Distrust*			
1	11	0.992	28 9 1 27 16 24 20 25 23 5
			17 18 22 8 21 12 2 19 6 4
			13 3 15 7 14
2	26	0.903	1 27 16 24 20 25 23 5 17 18
			22 8 21 12 2 19 6 4 13 3
			15 7 14
3	10	0.9	1 27 16 24 20 25 23 5 17 18
			22 8 21 12 2 19 6 4 13 3
			15 7 14
4	28	0.876	1 27 16 24 20 25 23 5 17 18
			22 8 21 12 2 19 6 4 13 3
			15 7 14
5	9	0.802	27 24 20 25 23 5 17 18 22 8
			21 12 2 19 6 4 13 3 15 7
			14
6	1	0.721	5 17 18 22 8 21 12 2 19 6
			4 13 3 15 7 14
7	27	0.688	18 8 21 12 2 19 6 4 13 3
			15 7 14
8	16	0.686	18 8 21 12 2 19 6 4 13 3
			15 7 14
9	24	0.677	18 8 21 12 2 19 6 4 13 3
			15 7 14
10	20	0.674	18 8 21 12 2 19 6 4 13 3
			15 7 14
11	25	0.655	18 8 21 12 2 19 6 4 13 3
			15 7 14
12	23	0.653	18 8 21 12 2 19 6 4 13 3
			15 7 14
13	5	0.605	21 12 2 19 6 4 13 3 15 7
			14
14	17	0.586	21 12 2 19 6 4 13 3 15 7
			14
15	18	0.532	12 2 19 6 4 13 3 15 7 14
16	22	0.518	19 6 4 13 3 15 7 14
17	8	0.503	2 19 6 4 13 3 15 7 14
18	21	0.439	19 6 4 13 3 15 7 14
19	12	0.413	19 4 13 3 15 7 14
20	2	0.342	4 13 3 15 7 14
21	19	0.298	4 13 3 15 7 14
22	6	0.278	13 3 15 7 14
23	4	0.168	13 3 15 7 14
24	13	0.051	7 14
25	3	0	7 14
26	15	−0.049	14
27	7	−0.127	
28	14	−0.173	
*Populism*			
1	27	1.12	26 28 1 11 16 9 24 20 21 18
			25 5 23 12 22 3 2 17 19 4
			13 8 15 6 14 7
2	10	1.049	26 28 1 11 16 9 24 20 21 18
			25 5 23 12 22 3 2 17 19 4
			13 8 15 6 14 7
3	26	0.87	16 9 24 20 21 18 25 5 23 12
			22 3 2 17 19 4 13 8 15 6
			14 7
4	28	0.859	16 9 24 20 21 18 25 5 23 12
			22 3 2 17 19 4 13 8 15 6
			14 7
5	1	0.803	9 20 21 18 25 5 23 12 22 3
			2 17 19 4 13 8 15 6 14 7
6	11	0.787	9 24 20 21 18 25 5 23 12 22
			3 2 17 19 4 13 8 15 6 14
			7
7	16	0.678	20 21 18 25 5 23 12 22 3 2
			17 19 4 13 8 15 6 14 7
8	9	0.612	20 21 18 25 5 23 12 22 3 2
			17 19 4 13 8 15 6 14 7
9	24	0.585	18 25 5 23 12 22 3 2 17 19
			4 13 8 15 6 14 7
10	20	0.463	5 23 12 22 3 2 17 19 4 13
			8 15 6 14 7
11	21	0.461	5 23 12 22 3 2 17 19 4 13
			8 15 6 14 7
12	18	0.417	23 12 22 3 2 17 19 4 13 8
			15 6 14 7
13	25	0.4	23 12 22 3 2 17 19 4 13 8
			15 6 14 7
14	5	0.304	23 12 22 3 2 17 19 4 13 8
			15 6 14 7
15	23	0.131	2 17 19 4 13 8 15 6 14 7
16	12	0.095	17 4 13 8 15 6 14 7
17	22	0.048	4 13 8 15 6 14 7
18	3	0	4 13 8 15 6 14 7
19	2	−0.047	4 13 8 15 6 14 7
20	17	−0.056	4 13 8 15 6 14 7
21	19	−0.068	4 13 8 15 6 14 7
22	4	−0.247	8 15 6 14 7
23	13	−0.293	8 15 6 14 7
24	8	−0.496	15 14 7
25	15	−0.675	
26	6	−0.703	
27	14	−0.716	
28	7	−0.839	

Note: This table presents an ordered listing ranging from high to low; the factor mean for each country is followed by the identification of countries having factor means that are statistically significantly different (*p* < 0.05). The numbers indicate the country code (see Table A2).
